# Review: Mesenchymal Stem Cell Therapy in Canine Osteoarthritis Research: “Experientia Docet” (Experience Will Teach Us)

**DOI:** 10.3389/fvets.2021.668881

**Published:** 2021-05-19

**Authors:** Carlien Brondeel, Glenn Pauwelyn, Evelien de Bakker, Jimmy Saunders, Yves Samoy, Jan H. Spaas

**Affiliations:** ^1^Department of Medical Imaging and Orthopedics of Domestic Animals, Faculty of Veterinary Medicine, Ghent University, Merelbeke, Belgium; ^2^Global Stem Cell Technology NV, Part of Boehringer-Ingelheim, Evergem, Belgium

**Keywords:** mesenchymal stem cell, osteoarthiritis, dog, autologous, allogeneic, xenogeneic

## Abstract

Osteoarthritis (OA) is currently an incurable and progressive condition in dogs causing chronic joint pain and possibly increasing disability. Due to the poor healing capacity of cartilage lesions that occur with OA, development of effective therapeutics is difficult. For this reason, current OA therapy is mostly limited to the management of pain and inflammation, but not directed ad disease modification. In the search for a safe and effective OA treatment, mesenchymal stem cells (MSCs) have been of great interest since these cells might be able to restore cartilage defects. The designs of OA studies on MSC usage, however, are not always consistent and complete, which limits a clear evaluation of MSC efficacy. The general study results show a tendency to improve lameness, joint pain and range of motion in dogs suffering from naturally-occurring OA. Assessment of the cartilage surface demonstrated the ability of MSCs to promote cartilage-like tissue formation in artificially created cartilage defects. Immunomodulatory capacities of MSCs also seem to play an important role in reducing pain and inflammation in dogs. It should be mentioned, however, that in the current studies in literature there are specific design limitations and further research is warranted to confirm these findings.

## Osteoarthritis

### Characteristics of Osteoarthritis

Osteoarthritis (OA) is a commonly seen condition in veterinary medicine, causing chronic pain and increasing disability due to progressive joint degeneration (1–5). Prevalence studies described that 2.5% of dogs presented to primary practices in the UK with OA and more than 20% of dogs over 1 year old in the US are affected by OA (1, 3, 5). Specific breeds (e.g., Labrador, Golden Retriever), castration, advanced age and obesity are suggested to be risk factors of OA development (1, 5, 6).

Although OA is often diagnosed in older dogs, it is not part of normal aging (4). Contrary to human and feline OA, canine OA is in general secondary to trauma, including abnormal loading on a normal joint (e.g., joint injury) or normal force on an abnormal joint (e.g., elbow and hip dysplasia) (1, 3, 4). Osteoarthritis is characterized by osteophyte formation, bone remodeling, changes in peri-articular tissue and synovitis (2, 3). Nevertheless, the main feature of OA is cartilage damage. When the cartilage homeostasis is disturbed, chondrocytes become “activated” and produce inflammatory mediators which are able to stimulate progression of cartilage damage and adjacent joint tissue alterations, establishing a vicious cycle of joint deterioration and worsening OA (3, 7).

Unfortunately, due to relative avascularity and therefore the lack of systemic regulation, the repair capacity of cartilage is very poor (2, 3). For this reason, OA is a life-long progressive disease that currently cannot be cured (4). The lack of an OA cure necessitates management to reduce pain and inflammation, to restore normal cartilage and joint function and to prevent further damage (2). Many therapeutics are available which all target different aspects of OA.

### Osteoarthritis Management

Pharmacologic analgesia is probably the most common component of OA management. Non-steroidal anti-inflammatory drugs (NSAIDs), tramadol and gabapentin are frequently used to reduce the pain and inflammation. However, a profound patient selection and follow-up is indicated since potential side effects are well-known (4, 5). Intra-articular (IA) administration of hyaluronic acid (HA) and platelet rich plasma (PRP) are effective to improve joint pain and mobility and to influence healing of bone, tendon, muscle and ligament, respectively (5, 8–11). It is also described that PRP induces chondrogenesis, is able to increase anti-inflammatory mediators and decrease pro-inflammatory mediators (11). Although effective to manage OA related complaints, HA and PRP are not able to cure cartilage damage. Since cartilage protection is a difficult task of OA management, various food supplementation products, frequently based on chondroitin, glucosamine, methylsulfonylmethane (MSM), glycosylated undenaturated type II collagen and omega-3 fatty acids, were developed. These nutraceuticals might act as “building blocks” of cartilage and reducers of inflammation. (4, 5, 12, 13). Unfortunately, except for undenaturated type II collagen and omega-3 fatty acids, scientific evidence on supplement efficiency is scares (4, 12–15). Alternative OA management includes physical rehabilitation and body weight control. Physiotherapeutic modalities and exercises reduce pain and improve movement and joint function (4, 5). A reduction of body weight decreases joint loading and the risk of joint injury (4–7, 12). Although this kind of OA management is simple, good results can only be achieved with owner compliance.

### Upcoming Importance of Mesenchymal Stem Cells in the Osteoarthritis Research Field

Since current OA management has its limitations and is not able to reverse cartilage damage, new and promising research areas have been explored. Biological therapies based on mesenchymal stem cells (MSCs) have become of great interest in both human and veterinary OA research. In contrast to embryonic stem cells, MSCs are derived from adult tissue (16). Being unspecialized and able to differentiate into multiple cell lineages, including chondrocytes, these adult stem cells have the capacity to repopulate cartilage defects (2, 17). Reduction of local and systemic inflammation can be enabled by the MSCs' immunomodulatory capabilities (2, 18, 19). Furthermore, MSCs apply paracrine signaling which stimulate local repair cells that may contribute to cartilage healing (2, 18–20). MSCs have also been shown to possess homing capacities, meaning they can be recruited, both locally and systemically, to sites of tissue injury (21).

In human research, study results strongly suggest that MSC therapy is effective in relieving pain and improving joint function in patients suffering from OA. Especially the effect on knee OA has been investigated thoroughly (22–24). Moreover, since no obvious adverse effects are described, MSC use in humans might be a safe and effective alternative for current OA management therapies (22, 23). In veterinary medicine, being humans' closest companions and susceptible to OA, dogs and horses have been the main focus of MSC research. As in human OA research, study results are promising, providing evidence that MSC treatments may be safe and effective (25, 26). However, both human and veterinary studies focused on the efficacy and safety of MSCs as an OA therapy, many features of MSCs are not yet completely understood. A growing knowledge about MSC capabilities may provide the solution for both human and veterinary patients suffering from OA by slowing down disease progression or even reversing the damage and restoring full function.

### Varieties of Mesenchymal Stem Cells in Veterinary Research

In mesenchymal stem cell research, three types of MSCs can be differentiated: autologous, allogeneic and xenogeneic MSCs. When a patient receives its own MSCs, these MSCs are autologous. Allogeneic MSCs refer to MSCs derived from a donor animal of the same species as the receiving animal. Application of donor MSCs of a different species is called xenogeneic. Autologous derived MSCs are preferably used in veterinary studies since these cells are immunologically compatible with the receiving patient (27). Also, use of autologous MSCs does not involve donor animal harvesting and so does not imply ethical issues (28, 29). Harvesting every single patient and producing autologous MSCs, however, is a time consuming and challenging task for most veterinary practices. For this reason, many research groups investigated MSCs derived from donor animals as an attractive alternative. Allogeneic and xenogeneic MSCs are “ready to treat” which means that they can be prepared and stored in commercial quantities (30). In donor-derived MSC studies, allogeneic MSCs are preferred since they are expected to have higher donor-host compatibility than xenogeneic MSCs (30). Nevertheless, some canine studies did investigate the application of xenogeneic equine and porcine MSCs (31, 32). Additional features of xenotransplantation are absence of canine-specific transferable pathogens and a higher culture capacity of equine compared to canine MSCs (19).

The possibility of allogeneic and even xenogeneic MSC usage was clarified by several canine and equine studies which described MSCs to be “immune privileged.” It is proposed that this interesting MSC feature is the consequence of an absent major histocompatibility complex (MHC) class II expression on its cell surface (27, 33). An absent MHC class II expression is favorable in allogeneic and xenogeneic circumstances since these molecules initiate an antigen-specific immune response by presenting extracellular pathogens to CD4^+^ T cells (34). More recent studies, however, described that MHC class II could be up-regulated *in vitro* when presenting the MSCs in inflammatory environments making them recognizable for the host's immune system. For this reason, this needs to be assessed before clinical use (27, 33). Others described the possibility of allogeneic and xenogeneic usage to be a consequence of immunomodulatory capabilities of MSCs which reduces the relevance of a cellular immune response after potential MHC upregulation. In addition, suppression of the host's immunological reaction toward donor MSCs might enable allo- and xenotransplantation (30).

To apply autologous, allogeneic or xenogeneic MSCs as a therapeutic tool, cells need to be harvested from tissue and cultured in laboratory conditions. In canine OA research, the use of a variety of MSC sources is described, such as adipose tissue (28, 32, 35–51), bone marrow (52–58), synovium (59), dental pulp (60), fetal adnexa (61), umbilical cord (62, 63) and peripheral blood (31). Currently, adipose tissue and bone marrow seem to be the most popular MSC sources. The popularity of adipose tissue-derived MSCs (AD-MSCs) can be attributed to an easy accessibility and expansion in culture (29, 64, 65).

The aim of the current review article is to provide a clear overview of currently reported canine OA research on MSC application, with a main focus on study execution, efficacy and safety results. Special attention is given to the distinctive features and challenges of autologous, allogeneic and xenogeneic MSC use.

## Mesenchymal Stem Cell Application in Canine Osteoarthritis Research

### Types of Canine Osteoarthritis Research on Mesenchymal Stem Cells

**Figure 1:**
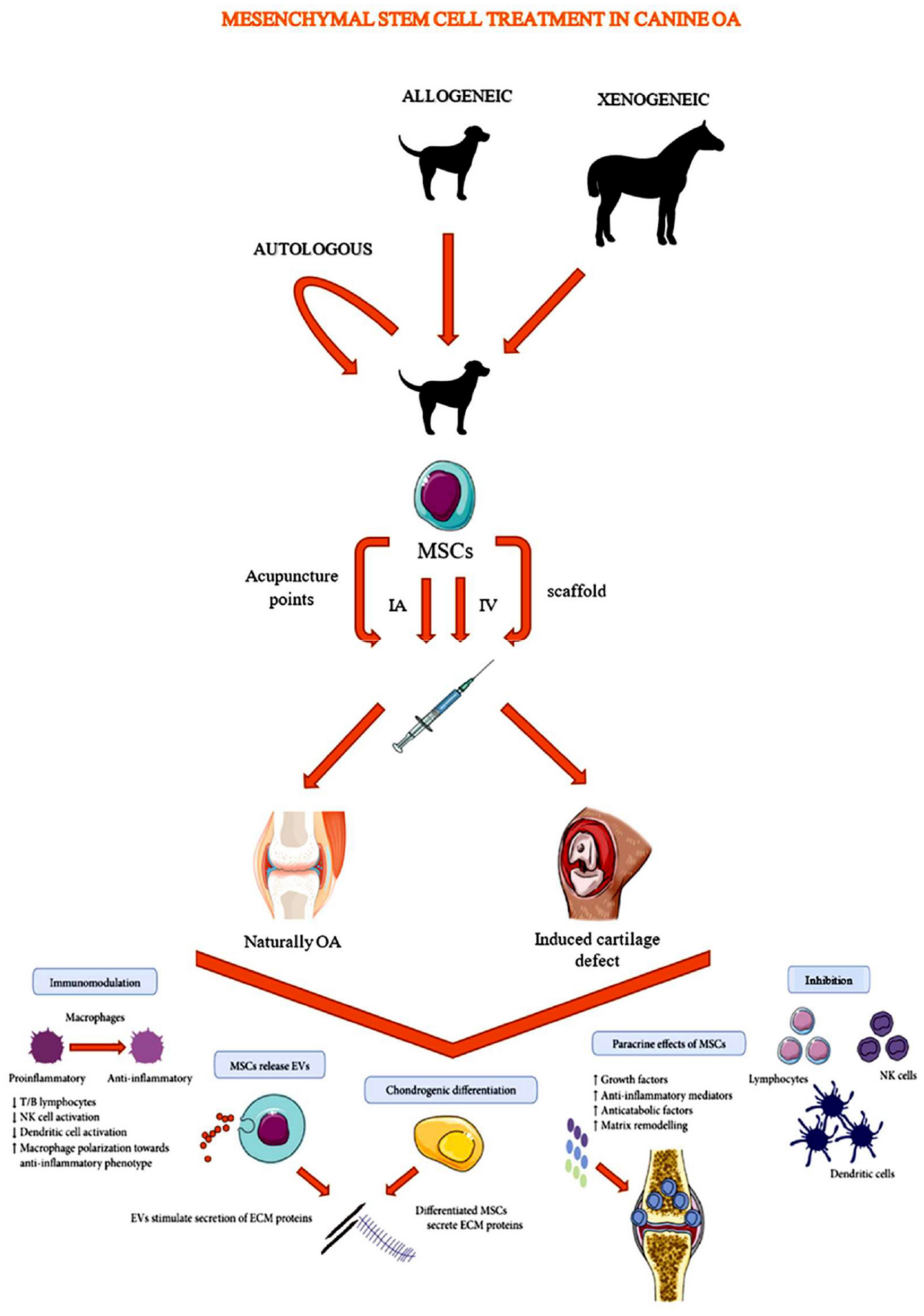
Schematic overview of mesenchymal stem cell administration in canine osteoarthritis studies [Modified from Lo Monaco et al. (20)].

Current canine OA research on auto-, allo-, and xenotransplantation of MSCs, can be subdivided into two study types (based on the study population) (Figure 1). The first type and majority of canine OA studies include companion animals suffering from naturally-occurring OA (Tables 1, 2, 3) (28, 31, 32, 35–50, 60–62). In studies of a second type, joint damage is induced in purpose-bred dogs in order to investigate the potential of MSCs to heal cartilage (Tables 4, 5) (51–59, 63).

**Table 1:** Chronologic presentation of studies on naturally occurring OA applying autologous MSC.

**Author**	**# Patients**	**Groups/treatment**	**OA location**	**MSC source**	**Injection site**	**Dosage MSC (cells)**	**# Injections**	**Outcome**	**Side effects MSCs**
Black et al., (28)	21	MSC: not specifiedControl: not specified	hip	fat	IA	4.2–5 × 10^6^	1	+	No
Black et al., (35)	14	MSC: 14	elbow	fat	IA	3–5 × 10^6^	1	+	No
Guercio et al., (37)	4	MSC + HA: 2MSC + PRP: 2	elbow	fat	IA	3–5 × 10^6^	1	+	No
Yoon et al., (50)	1	MSC + surgery + HA: 1	stifle	fat	IA	1 × 10^6^	1	+	No
Vilar et al., (48)	13	MSC + PRP: 8Control: 5	hip	fat	IA	15 × 10^6^	1	+	No
Cuervo et al., (36)	35	MSC: 18PRP: 17	hip	fat	IA	30 × 10^6^	1	+	No
Nicpon et al., (42)	12	MSC: 8Control: 4	elbow	fat	IA	1.5 × 10^6^	1	+	No
Vilar et al., (46)	15	MSC: 10Control: 5	hip	fat	IA	15 × 10^6^	1	+	Yes (1 dog)
Mohoric et al., (41)	10 (20 stifles)	MSC: 10 stiflesControl: 10 stifles	stifle	fat	IA	2–3 × 10^6^	1	+	No
Vilar et al., (47)	15	MSC: 10Control: 5	hip	fat	IA	15 × 10^6^	1	+	No
Srzentic Drazilov et al., (45)	10	MSC: 10	Different joints	fat	IA	>15 × 10^6^	1	+	No

**Table 2:** Chronologic presentation of studies on naturally occurring OA applying allogeneic MSC.

**Author**	**# Patients**	**Groups/treatment**	**OA location**	**MSC source**	**Injection site**	**Dosage MSC (cells)**	**# Injections**	**Outcome**	**Side effects MSCs**
Marx et al., (40)	9	MSC: 5Stromal vascular fraction (SVF): 4	hip	fat	3 acupuncture points	0.2–0.8 × 10^6^	1	+	No
Bootcha et al., (60)	8	MSC: 8	hip	Dental pulp	IA	5 × 10^6^	Multipe (not specified)	+	No
Harman et al., (38)	74	MSC: 38Control: 36	Different joints	fat	IA	12 × 10^6^	1	+	No
Kriston-Pal et al., (39)	30 (39 elbows)	MSC + HA: 30	elbow	fat	IA	12 × 10^6^ ± 3.2 × 10^6^	1	+	Yes (2 of 39 joints)
Shah et al., (44)	203	MSC IA: 128MSC IV: 65MSC IA + IV: 10	Different joints	fat	IA and/or IV	Not specified	1	IA/IA+IV: 90% +IV: 76% +	Yes (2 dogs)
Cabon et al., (61)	22	MSC: 22Repeated treatment (RT): 8	Different joints	Fetal adnexa	IA	≥ 10 × 10^6^	1 2 (8 dogs)	+	MSC: 5 <48u RT: 4 <48u
Kim et al., (62)	68	MSC: 38Control: 30	elbow	Umbilical cord	IA	7 × 10^6^	1	+	No
Olson et al., (43)	13	MSC: 13	elbow	fat	IV	1–2 × 10^6^/kg body weight	3	+ (subjective)= (objective)	No
Wits et al., (49)	12 (24 hips)	MSC: 4MSC + HA: 4Control: 4	hip	fat	IA	5 × 10^6^	1	=	No

**Table 3:** Chronologic presentation of studies on naturally occurring OA applying xenogeneic MSC.

**Author**	**# Patients**	**Groups/treatment**	**OA location**	**MSC source**	**Injection site**	**Dosage MSC (cells)**	**# Injections**	**Outcome**	**Side effects MSCs**
Tsai et al., (32)	3	MSC: 3	stifle	Porcine fat	IA	5 × 10^6^	1	+	No
Daems et al., (31)	6	MSC: 6Control: 6 (same dogs, 6 weeks before MSC)	elbow	Equine peripheral blood	IA	1 × 10^6^	1	+ (owner)= (objective)	No

**Table 4:** Chronologic presentation of model based studies applying autologous MSC.

**Author**	**# Patients**	**Groups/treatment**	**Type defect**	**Location cartilage defect**	**MSC source**	**Injection site**	**Dosage MSC (cells)**	**# Admini-strations**	**Outcome**	**Side effects MSCs**
Mokbel et al., (55)	32	MSC 1 day after defect: 12MSC 1 month after defect: 12Control: 8	Partial thickness cartilage	Lat. condyle femur	Bone marrow	IA	7–8 × 10^6^	1	+	No
Qiang et al., (56)	12	MSC + scaffold: 8Control: 4	Osteo-chondral	Bilateral condyle femur	Bone marrow	Implantation with scaffold	1 × 10^6^	1	+	No
Miki et al., (59)	24 (48 stifles)	0 MSCs + 0% HA: 3 stifles 5 × 10^5^ MSCs + 0.01% HA: 3 stifles 5 × 10^6^ MSCs + 0.1% HA: 3 stifles 5 × 10^7^ MSCs + 0.5% HA: 3 stifles 0 MSCs + 0.5% HA: 3 stifles 5 × 10^5^ MSCs+ 0% HA: 3 stifles 5 × 10^6^ MSCs+ 0.01% HA: 3 stifles 5 × 10^7^ MSCs + 0.1% HA: 3 stifles 0 MSCs + 0.1% HA: 3 stifles 5 × 10^5^ MSCs + 0.5% HA: 3 stifles 5 × 10^6^ MSCs+ 0% HA: 3 stifles 5 × 10^7^ MSCs+ 0.01% HA: 3 stifles 0 MSCs+ 0.01% HA: 3 stifles 5 × 10^5^ MSCs + 0.1% HA: 3 stifles 5 × 10^6^ MSCs + 0.5% HA: 3 stifles 5 × 10^7^ MSCs+ 0% HA: 3 stifles	Partial thickness cartilage	Med. condyle femur	synovium	IA	See column group/treatments	1	+	No
Yamasaki et al., (58)	15 (30 stifles)	MSC + serum + HA: 15 stifles Serum + HA: 15 stifles	Full thickness cartilage	Patellar groove	Bone marrow	Direct implantation in defect	0.2–2.8 × 10^7^	1	+	No
Kazemi et al., 2017 (53)	12 (24 stifles)	MSC + PRF: 12 stifles Control: 12 stifles	Osteo-chondral	Med. condyle femur	Bone marrow	Direct implantation in defect	1 × 10^6^	1	+	No

**Table 5:** Chronologic presentation of model based studies applying allogeneic MSC.

**Author**	**# Patients**	**Groups/treatment**	**Type defect**	**Location cartilage defect**	**MSC source**	**Injection site**	**Dosage MSC (cells)**	**# Admini-strations**	**Outcome**	**Side effects MSCs**
Duan et al., (52)	12 (24 stifles)	MSC + scaffold: 8 stifles Scaffold: 8 stifles Control: 8 stifles	Osteo-chondral	Patellar groove	Bone marrow	Implantation with scaffold	1 × 10^4^	1	+	No
Qiang et al., (57)	12 (24 hips)	MSC + scaffold: 12 hipsControl: 12 hips	Osteo-chondral	Femoral head	Bone marrow	Implantation with scaffold	Seeded with 5 × 10^7^/mL	1	-	No
Yun et al., (51)	24	MSC: 6PRP: 6MSC + PRP: 6Control: 6	Cranial cruciate ligament	/	fat	IA	1 × 10^7^	4	+	No
Li et al., (54)	24 (48 stifles)	MSC + HA: 8HA: 8Control: 8	Partial thickness cartilage	Bilateral condyle femur + patellar groove	Bone marrow	IA	1 × 10^7^	1	+	No
Zhang et al., (63)	8	MSC: 4Control: 4	cartilage	Condyle femur + patella	Umbilical cord	IA	1 × 10^6^	2	+	No

Studies evaluating naturally-occurring OA have an advantage over model-based studies since they evaluate the effect of MSCs on a real world (field) condition (28, 31, 32, 35–50, 60–62). Nevertheless, establishing natural OA studies often encounters some difficulties. Since, in this type of study, the targeted population consists of owned animals, patient recruitment is challenging and study populations are often rather small (31, 32, 37, 40–42, 45, 49, 50, 60). Also, a control group is frequently missing because owners want their dog to actually be treated (32, 35, 37, 39, 43, 45, 60). Moreover, OA affected dogs often have problems in multiple joints and the severity of OA can differ, which may complicate study outcome interpretation (62).

In studies based on OA models, cartilage or cranial cruciate ligament defects are created surgically in joints of purpose-bred dogs before MSC administration (51–59, 63). In some studies, however, MSCs are administered during or immediately after surgery, which might not adequately represent the effect on chronic OA (52, 53, 56–58). Compared to studies with client owned animals, model based studies can easily include control groups and provide the opportunity to apply a wider variety of research tools (e.g., histopathology) (32). Unfortunately, to enable histo- and pathologic examinations, which are essential for thorough safety assessment, animal sacrifice is sometimes required.

### Mesenchymal Stem Cell Administration in Osteoarthritis Studies

Several administration routes have been investigated to administer MSCs to an OA affected or a purpose-bred dog (Figure 1). Mostly, MSCs are administrated directly into the affected joint by an IA injection (28, 31, 32, 35–39, 41, 42, 44–51, 54, 55, 59–63). However, since MSCs are known to have homing capacities, some studies investigated the effectiveness of other administration routes (21, 40, 43, 44) such as: intravenous (IV) administration (43, 44) and administration via acupuncture points (40). Systemic delivery may be advantageous since these MSCs may evoke a greater interaction with the immune system than local delivered MSCs (43, 66). MSCs seem to be able to enhance white blood cell activity, differentiation and migration to lesion sites (66). Although further research is indicated to clarify the exact mode of action, such a systemic interaction with the immune system may lead to reduced inflammation at multiple affected joints in the body (43, 66). Moreover, IV administration can make MSC based treatments more accessible for primary practice. Acupuncture point administration might, according to a study in rats and dogs, enhance blood flow and increase the level of vascular endothelial growth factor (VEGF), transforming growth factor (TGF)-β1 and nitric oxide (NO), improving angiogenesis and arteriogenesis (40, 67). Other studies described the deposition of MSCs into the joint using a scaffold. Scaffolds are tissue-engineered constructs, consisting of a distinct cartilage and bone layer, which can be transplanted into large osteochondral defects to offer a template for new tissue formation and organization. Prior to transplantation, MSCs are seeded on these scaffolds (52, 56, 57). Based on a matched-pair study by Kim et al. (68), MSC administration on a scaffold was more efficient to improve clinical and arthroscopic outcomes in humans with stifle OA than MSC administration via IA injection (68). However, since the wide variety of described scaffold types, the finding of Kim et al. (68) should not be generalized.

In current OA research on MSCs, specific dose determination and safety studies are currently lacking and so the amount of MSCs and frequency of administration is very variable between publications. Doses are often based on the knowledge and experience of the authors, the applied tissue, the laboratory conditions, the amount of passages and viable cells. In autologous studies evaluating naturally-occurring OA, AD-MSCs dose varied from 1 × 10^6^ to >15 × 10^6^ cells administered with a single IA injection (28, 35–37, 41, 42, 45–48, 50). Doses of single and repeated IA, IV and acupuncture point injections of allogeneic MSC to OA affected dogs ranged from 0.2 × 10^6^ cells to 2 × 10^6^ cells per kilogram bodyweight (38–40, 43, 44, 49, 60–62). In xenogeneic naturally-occurring OA studies, single IA injections of 5 × 10^6^ porcine MSCs in canine stifle joints (32) and 1 × 10^6^ equine MSCs in canine elbow joints (31) were performed. In model-based autologous studies, the amount of MSCs administered by a single IA injection or implantation ranged from 5 × 10^5^ to 5 × 10^7^ cells (53, 55, 56, 58, 59). To transfer the MSCs to the defect side in allogeneic studies, up to 5 x 10^7^ MSCs were seeded on a scaffold (52, 57) and 1 × 10^6^ to 1 × 10^7^ MSCs were injected IA (up to four injections) (51, 54, 63).

Within the field of MSC research, MSCs are often characterized before administration. Next to the standard measures such as cell count, viability, sterility and cell adhesion, MSCs can be characterized using different cell surface markers, trilineage differentiation and morphology characterization. However, the majority of canine OA studies using MSCs did not perform a complete characterization of their applied cells. In most studies on naturally-occurring OA in dogs, at least two of the earlier mentioned analysis/analyses of MSC characteristics were lacking. The types of lacking analyses differed greatly between studies, but in general cell morphology and proliferation were the least evaluated MSC characteristics. An analyzation of the cell surface markers was described most commonly (28, 32, 35–50, 60–62). Daems et al. (31), who investigated the use of xenogeneic MSCs in dogs with naturally occurring OA, presented the most complete set of MSC characterizing analyses (31). In canine model-based OA studies on MSC usage, the lack of performed MSC characterizing analyses was even more pronounced than in naturally-occurring OA studies (51–59, 63). In most of these studies, only an evaluation of cell adhesion and cell surface markers was performed (51, 53, 54, 63). This insufficient evaluation of MSC characteristics is an important limitation in current canine MSC research, making proper data interpretation and comparison challenging. To improve future studies, MSC characterizing analyses need to become standardized.

Besides the MSCs, some research groups administrated additional products and evaluated their possible MSC potentiating effect in OA affected joints. Typically, the additional products were HA (37, 39, 49, 50, 54, 58, 59) or PRP (36, 37, 48, 51, 53). A comparison between the clinical effect of solo administration of additional products and MSCs was, although interesting, only made by three research groups (36, 54, 59).

### Mesenchymal Stem Cell Efficacy Evaluation in Osteoarthritis Studies

Evaluation of MSC efficacy as an OA therapy was conducted by a wide array of research tools. In general, studies evaluating naturally-occurring OA were evaluated orthopedically based on lameness, pain at joint manipulation and/or range of motion (ROM) (28, 31, 32, 35, 36, 38–42, 44, 45, 49, 60–62). Additionally, assessment of the effect of an MSC treatment on OA affected dogs may include owner questionnaires/evaluations (28, 31, 32, 35–41, 43, 47, 60–62), medical imaging (31, 32, 36, 41, 42, 49, 60), synovial fluid analyses (31, 41–43) and objective gait analysis by force or pressure plate (31, 32, 43, 46–48, 62). In most naturally-occurring OA studies, MSC safety was also assessed (31, 32, 36, 38, 42, 43, 49, 61, 62). By using purpose-bred dogs instead of client-owned dogs, model-based OA studies allowed evaluation of MSC treatments with macroscopic and/or microscopic joint assessments (51–59). Additional applied research techniques/tools described in autologous and allogeneic model-based studies were: lameness evaluations (51, 55), medical imaging (54, 57, 63), biochemical evaluations (56), biomechanical evaluations (51, 56, 57), micro-computer tomography (CT) (52, 56, 57), green fluorescent protein (GFP)-labeling for homing assessment (55), real-time polymerase chain reaction (PCR) (51), spectrophotometry (51), scanning electron microscopy (SEM) (63) and blood analyses (63).

## Study Outcomes

### Lameness and Joint Function

Study outcomes based on lameness, pain at joint manipulation and/or range of motion were favorable for all MSC types, sources and administration routes, and in both naturally-occurring OA and model based studies (28, 31, 32, 35–42, 44–48, 50, 51, 55, 60–62). For example, after a single IA injection of autologous adipose tissue-derived MSCs to 10 OA affected dogs, significant improvement of lameness and ROM up to 4 years post-treatment was described (45). Compared to IA administration, studies on allogeneic adipose tissue-derived MSCs showed IV administration to be clinically less satisfying (43, 44). In Australia, allogeneic adipose tissue-derived MSCs have been commercially available since 2010 as IA and IV treatmentsfor dogs suffering from OA. Shah et al. (44) reported these treatment results and showed a higher percentage of dogs to have a good to excellent quality of life (based on pain, mobility and daily activity) after IA or combined IA-IV treatment (~90%) than after IV treatment alone (76%) (44). Olsen et al. (43) reported improvement of client-specific outcome measures, however, objective outcome measures did not confirm these results (43). One study on allogeneic adipose tissue-derived MSCs described clinical improvement after acupuncture point injections in four of five dogs suffering from hip OA (40). Addition of PRP and HA to MSC treatments was reported to have a positive effect on study outcomes (37, 39, 49, 50). Nevertheless, only one study made a comparison of MSC treatment with and without additional product (49). Wits et al. showed a greater/earlier improvement of lameness and pain at joint manipulation after combined IA administration of allogeneic adipose tissue-derived MSCs and HA than after solo IA administration of MSCs (49). The difference in effect of additional or solo administration of HA or PRP was addressed by three research groups. Two model based studies describe a better cartilage repair after combined IA administration of autologous synovium and allogeneic bone marrow derived MSCs and HA than after HA alone (54, 59). A study that investigated the joint function after a single IA injection of autologous adipose tissue-derived MSCs and a single IA injection of PRP found enhanced joint mobility and functionality for both products. For these parameters, however, the effect of PRP was less pronounced or did not last as long as the effect of the MSCs (36).

Gait analyses based on force or pressure plate enables objective evaluation of a dog's lameness and limb function. Nevertheless, only a few research facilities studying the effect of autologous, allogeneic and xenogeneic MSCs evaluating naturally-occurring OA were equipped with a force or pressure plate (31, 32, 43, 46–48). The objective gait evaluations, however, did not always support clinical lameness exam outcomes. For example, Vilar et al. (46, 47). demonstrated significant improvement of mean peak vertical force (PVF) and vertical impulse (VI) values within the first 3 months after solo IA adipose tissue-derived MSC treatment (46, 47). Despite subjective assessment showing improvement, the dogs returned to the initial lameness state 6 months post-treatment (47). Others did not report any significant change in gait evaluation (31, 32, 43, 62). One study on autologous adipose tissue-derived MSCs reported a reduced lameness based on force plate analyses after a combined MSC and PRP administration (48). Compared to similar studies of the same research group only using MSCs, addition of PRP seems to enable a more long-lasting lameness reduction (46–48).

MSC treatments seem to have a favorable clinical effect on lameness, joint pain and ROM. Although IA administration appears to be most promising, careful data interpretation is indicated since clinical lameness results are not always supported by objective gait analysis. Additional administration of HA and PRP may improve both clinical and pressure/force plate outcomes.

### Osteoarthritis Progression

None of the studies on autologous and allogeneic MSC in dogs with naturally-occurring OA involving radiographic and CT imaging did show improvement of superficial bone changes 3 – 12 months post-treatment (36, 41, 42, 49, 60). Nevertheless, throughout the study period, no radiographic OA progression was detected after IA application of autologous and allogeneic adipose tissue-derived MSCs (41, 49). In contrast, a study applying autologous adipose tissue-derived MSCs in combination with HA and patellar luxation surgery described a decrease in osteophytes and subchondral cystic lesions on radiographic evaluation (50).

Based on radiographic and CT imaging, it was concluded that therapeutically administered MSCs are probably unable to reverse OA-related superficial bone changes, however, they may be able to slow down or even stop OA progression. Additionally, it is important to realize that bone remodeling and thus radiographic changes take time, which might have been the limiting factor of some of these studies.

### Cartilage and Subchondral Bone Evaluation and Properties

The presence of newly formed cartilage was visualized by macroscopic, microscopic and arthroscopic assessment and by MRI and SEM screening. In several model based studies evaluating autologous or allogeneic MSC usage, macroscopic and histologic evaluations, conducted between 8 and 24 weeks after MSC treatment, showed that experimentally created cartilage defects were (partially) filled with cartilage-like tissue, while the defects of the control groups were filled with fibrous tissue (52–56, 58, 59). An arthroscopic evaluation and biopsy of an natural occurring OA defect showed cartilage regeneration 12 months after combined IA administration of allogeneic adipose tissue-derived MSCs and HA (39). In model-based studies on IA administration of allogeneic umbilical cord-derived and bone marrow-derived MSCs, respectively joint repair after 28 days and the presence of cartilage-like tissue after 28 weeks were reported based on MRI screening (54, 63). Li et al. (54) described cartilage-like tissue to be present after combined administration of MSCs and HA and after administration of HA alone, however, the cartilage-like tissue was thicker after the combined administration (54). To obtain a highly detailed presentation of the cartilage surface, SEM was applied by a model-based study on allogeneic umbilical cord-derived MSC usage. In the treated group, which was IA injected with MSCs, some small protuberances of cartilage on the articular surface were present 35 days post-treatment. The thickness of the new cartilage in the treated group was significantly higher than the thickness of the cartilage in the control group (63).

Cartilage production was assessed by a research group evaluating allogeneic transplantation of adipose tissue-derived MSCs in a cranial cruciate ligament transection study. The research group analyzed immunoreactivity against BrdU, a cell proliferating marker. The number of BrdU-positive cells, proliferating chondrocytes, was significantly decreased in the control group and significantly increased in the treatment groups. The increase of cell proliferation was most significant after a combined treatment of MSCs and PRP (51). Additionally, an immunohistochemistry test against caspase-3 and poly adenosine diphosphate–ribose polymerase (PARP), which play a role in cell apoptosis and death, respectively, was conducted. The test results showed an increase of caspase-3- and PARP-positive cells in OA conditions and a decrease after MSC and/or PRP administration (51). Evaluations based on real-time PCR demonstrated an increase of extracellular matrix (ECM)-related genes (cartilage aggrecan and sex-determining region Y-related high mobility group-box [SOX]9) in the groups treated with MSCs, PRP or a combination. The increase of ECM-related genes was most prominent after the combination treatment (51).

The content of collagen and glycosaminocglycan (GAG), important components of hyaline cartilage, were evaluated by biochemical, spectrophotometric and immunochemical analyses. Biochemical evaluations performed by a research group evaluating autologous bone marrow-derived MSCs in OA models, demonstrated the GAG content of tissue-engineered cartilage to be 84.82% 6 months after MSC enriched scaffold implantation. This reported percentage approaches the GAG content of normal cartilage *in vivo* (56). Spectrophotometric analyses of the ECM were performed by a cranial cruciate ligament transection study on allogeneic adipose tissue-derived MSCs and showed the collagen and GAG content to be significantly higher in the MSC treatment group than in the control group (51). Immunochemical analyses conducted by a model-based study on allogeneic bone marrow-derived MSC usage demonstrated that type II collagen formation was present in a group with MSC administration, but higher in another group with combined MSC and HA administration. The study suggested that combined usage of MSCs and HA could stimulate the regeneration of cartilage better than HA alone (54).

In model-based studies on autologous or allogeneic bone marrow-derived MSC application, implanting scaffolds in osteochondral defects, subchondral bone was assessed by micro-CT (52, 56, 57). Qiang et al. (56) showed regularly formed mature trabecular bone 3 and 6 months after autologous MSC enriched scaffold treatment. Between control and treatment group, however, no significant differences on micro-CT assessment were found (56). On micro-CT evaluations of Duan et al. (52), large quantities of spongy bone were seen in the pours of the scaffold, both in the MSC enriched scaffold group and in the unseeded scaffold group (52). In contrast, in the study of Qiang et al. (57), micro-CT showed collapse of the high-load-bearing areas of the femoral head. Furthermore, the bone volume fraction (BVF) was lower in the treated than in the normal femoral heads (57).

Biomechanical analyses of cartilage and subchondral bone were conducted in model-based studies after autologous and allogeneic MSC administration (51, 56, 57). Biomechanical analyses applying autologous bone marrow-derived MSCs tested the stiffness of cartilage and osteochondral bone after MSC enriched scaffold implantation. 6 months after treatment, cartilage stiffness was 70.77% of normal cartilage and osteochondral bone stiffness was 74.95% of normal osteochondral bone. Thus, the newly formed cartilage and subchondral bone were only slightly softer than normal osteochondral tissue (56). In an allogeneic transplantation study, the focal compression strength of the affected articular surface was, compared to the control, significantly higher 5 months after administration of adipose tissue-derived MSCs and/or PRP. The highest strength was seen in the group combining MSCs and PRP (51). Reported stiffness of the high-load-bearing area of the femoral head 3 and 6 months after allogeneic bone marrow-derived MSC enriched scaffold treatment was only 57.3% of normal stiffness. In this study, the scaffold failed to repair the created osteochondral defects (57).

Although study protocols are very variable, according to visual evaluations of the joint surface and assessments of cartilage production, MSC administration seems to be sufficient to cover chondral defects with cartilage-like tissue. The compositional and biomechanical characteristics of this newly formed cartilage are very similar to those of *in vivo* cartilage.

### MSC Homing

MSC homing capacities were assessed by a model-based study on autologous bone marrow-derived MSCs, which performed a fluorescence analysis. GFP-labeled MSCs were detected in neocartilage 2 and 8 weeks after IA injection and thus confirmed MSC homing (55). These results are comparable to a similar model-based study in donkeys assessing the homing capacity of IA injected, GFP-labeled autologous bone marrow-derived MSCs. Fluorescence microscopy assessment confirmed the incorporation of GFP-labeled MSCs in the newly formed cartilage 1, 2 and 6 months post-treatment (69).

In contrast, in a study on IV injection of allogeneic adipose tissue-derived MSCs, MSCs labeled with a cell membrane dye were rarely detected in the synovial fluid (43). In a rabbit and horse study, however, homing of IV injected, respectively, autologous and allogeneic MSCs to places of tissue injury was confirmed (70, 71). Olsen et al. (43) reported pulmonary trapping, to early assessment of the synovial fluid, detainment in the synovial membrane and inadequate cell labeling as possible causes of their insufficient MSC homing detection (43).

Based on labeled MSCs detection studies, the principle of MSC homing after IA and IV administration seems to be promising for OA affected animals. In dogs, however, there is a discrepancy between study results after IA and IV MSC administration, which is probably a consequence of MSC distance to injury location. Otherwise, insufficient homing detection may be caused by unfitted assessment procedures. To uncover assessment failures, study results should be compared to clinical outcomes. For a better understanding of MSC homing in dogs, further research is warranted.

### Inflammation Biomarkers and Synovial Fluid Characteristics

Evaluations of synovial fluid were performed in naturally-occurring OA studies (31, 41–43). A decrease of chronic inflammation signs was detected 6 months after IA administration of autologous adipose tissue-derived MSCs. The numbers of leucocytes, neutrophils and mononuclear cells were within normal ranges, as was the color and viscosity of the synovial fluid (42). 12 months after autologous adipose tissue-derived MSC administration, however, another study did not detect significant synovial fluid changes (41). Also, after repeated IV injection of allogeneic adipose tissue-derived MSCs, no significant changes in OA biomarkers [prostaglandin E2 (PGE2) and matrix metalloproteinase-2 (MMP-2)] were found (43). After IA administration of equine peripheral blood-derived MSCs, analysis of synovial fluid, focusing on hemarthrosis and viscosity, remained unchanged in dogs suffering from naturally-occurring elbow OA (31).

Immunohistochemistry was applied in a cranial cruciate ligament transection study on allogeneic transplantation of adipose tissue-derived MSCs. Immunoreactivities in the cartilage tissue were determined against pro-inflammatory cytokines, tumor necrotic factor (TNF)-α, cyclooxygenase (COX)-2, interleukin (IL)-1β, inducible nitric oxide synthase (iNOS) and interferon (IFN)-γ, which significantly decreased in the treatment group compared to the control group (51).

In a model-based study, blood analyses after IA allogeneic umbilical cord-derived MSC treatment evaluated inflammatory factors such as IL-6, IL-7 and TNF-α. No significant differences were detected 3 and 28 days after MSC treatment. At day seven, however, IL-6 and TNF-α were significantly higher in the untreated group. At day fourteen, significantly lower levels of IL-6, IL-7 and TNF-α were detected in the treated group (63).

According to blood analyses and immunoreactivity evaluations of the cartilage surface, MSC administration appears to decrease levels of inflammatory factors. Assessments of synovial fluid, however, did not always show significant changes. Evaluation of the effect of MSCs on joint inflammation is challenging due to the variety of study designs and assessment protocols. The applied MSC type, for example, may influence study results. To better understand the effect of MSC treatments on joint inflammation, further investigation is indicated.

### Safety

A safety assessment of IA and IV administration of MSCs was performed in some naturally-occurring OA studies, but important adverse events were not reported (31, 32, 36, 38, 42, 43, 49, 61, 62). Only a few minimal side effects were noted by a minority of studies, such as worsening of lameness due to injection difficulties (46), mild skin allergy (44) and self-limiting joint distension (39, 61). Also, no MSC related negative effects were reported in model-based studies (51–59, 63).

## Prospectives and Considerations of MSC Based OA Therapies

Therapies based on MSCs seem to be promising for improvement in joint function and to heal cartilage defects in OA affected dogs. MSCs have the ability to forge a novel means to manage not only the clinical impact of OA in dogs, but also to modulate the disease. To overcome the practical difficulties of harvesting and cultivating autologous MSCs, “ready to treat” products based on allo- and xenotransplantation would be ideal for the practitioner. Moreover, to facilitate MSC administration, systemic delivery features are of researchers' interest.

In recent literature, suggestions have been made about other interesting stem cell related therapies besides MSC-based therapies. Induced pluripotent stem cells (iPSCs) can be obtained by reprogramming adult cells and have the ability to differentiate into any cell type of the body. Compared to MSCs, iPSCs have a greater differentiation potential and might be able to provide a higher stem cell yield per donor. However, iPSCs are described to be related to tumorigenesis, thus making them less safe to use and unfitted as an MSC alternative (72–74). To be able to consider iPSCs in future medicine, additional studies addressing safety and efficacy need to be performed. Alternative upcoming research has been directed toward cell-free stem cell related therapies. Cell-free stem cell related research focuses on the stem cell's paracrine factors, including extracellular vesicles (EVs), which seem to play an important role in its effectiveness. It is hypothesized that these EVs may be able to heal and prevent tissue damage with a lesser impact on the immune system. Nevertheless, before considering this MSC alternative, more studies need to be conducted according to mode of action, bioavailability and administration of EVs (26, 74).

## Conclusion

In current canine medicine, a variety of MSC studies were enrolled to encounter the problem of OA. These studies have were very different based on study design, e.g., MSC sources, MSC dosage, administration and efficacy evaluation. However, in general, studies on auto-, allo-, and xenotransplantation of MSCs show to be promising. Research assessing the effect of MSCs on naturally occurring OA mostly demonstrated positive clinical outcomes for all three transplantation types, e.g., a decrease of lameness and joint pain and an increase of joint function. The reported clinical signs were most significant after IA MSC administration. In contrast, results based on medical imaging, objective gait analysis and synovial fluid evaluations were more doubtful. Addition of PRP or HA might be able to improve treatment outcome compared to solo MSC administration. In accordance with studies on naturally occurring OA, model-based studies administering autologous or allogeneic MSCs described reduced lameness and joint discomfort. Moreover, a variety of research tools showed that the administration of MSCs, whether or not on a scaffold, did induce the formation of cartilage-like tissue. Both in naturally-occurring OA and model based studies, the limited adverse events were minor, indicating that MSCs can be applied safely in canine OA patients.

Although many promising results of MSC studies, careful data interpretation is indicated since the reported study set-ups are often very different which makes outcome evaluation and comparison challenging. Also, naturally-occurring OA studies are frequently lacking sufficient study populations and/or control groups, depreciating reported findings. To overcome these hurdles, standardization should be provided by future development of evidence based protocols. Such protocols should ensure strictly designed, blinded, randomized and controlled studies applying well-characterized MSCs (i.e., determination of cell viability, morphology, presence or absence of cell surface markers, differentiation and population doubling times) at considerable dosages. Moreover, further research investigating mode of action and safety will attribute to a better understanding of the possibilities of MSCs as an OA healing product.

## Author Contributions

Data was gathered by CB. The final paper was produced by CB under direct supervision of GP. Intellectual support was provided by JS, YS, and EB. All authors contributed to the article and approved the submitted version.

## Conflict of Interest

JS is employed with Boehringer-Ingelheim Animal-health (BI-AH) and author GP is employed by Global Stem cell Technology (GST). The remaining authors declare that the research was conducted in the absence of any commercial or financial relationships that could be construed as a potential conflict of interest.

## Abbreviations

AD-MSCs: adipose tissue-derived MSCs
BVF: bone volume fraction
COX: cyclooxygenase
CT: computer tomography
ECM: extracellular matrix
ePB-MSCs: equine peripheral blood derived mesenchymal stem cells
EVs: extracellular vesicles
GAG: glycosaminoglycan
GFP: green fluorescent protein
HA: hyaluronic acid
IA: intra-articular
IL: interleukin
iNOS: inducible nitric oxide synthase
iPSCs: induced pluripotent stem cells
IFN: interferon
IV: intravenous
MHC: major histocompatibility complex
MRI: magnetic resonance imaging
MSCs: mesenchymal stem cells
MSM: methylsulfonylmethane
NO: nitric oxide
NSAIDs: non-steroidal anti-inflammatory drugs
OA: osteoarthritis
PARP: poly adenosine diphosphate–ribose polymerase
PCR: polymerase chain reaction
PRP: platelet rich plasma
PVF: peak vertical force
ROM: range of motion
RT: repeated treatment
SEM: scanning electron microscopy
SOX: sex-determining region Y-related high mobility group-box
TENS: transcutaneous electrical nerve stimulation
TNF: tumor necrotic factor
TGF- β1: transforming growth factor-β1
US: ultrasound
VEGF: vascular endothelial growth factor
VI: vertical impulse.
